# Comparative Label-Free Liquid Chromatography–Mass Spectrometry Milk Proteomic Profiles Highlight Putative Differences between the Autochthon Teramana and Saanen Goat Breeds

**DOI:** 10.3390/ani13142263

**Published:** 2023-07-10

**Authors:** Alessio Di Luca, Francesca Bennato, Andrea Ianni, Lisa Grotta, Michael Henry, Paula Meleady, Giuseppe Martino

**Affiliations:** 1 Department of Bioscience and Technology for Food Agro-Food and Environmental Technology, University of Teramo, 64100 Teramo, Italy; adiluca@unite.it (A.D.L.); fbennato@unite.it (F.B.); aianni@unite.it (A.I.); lgrotta@unite.it (L.G.); 2 National Institute for Cellular Biotechnology, Dublin City University, Dublin 9, Dublin, Ireland; michael.henry@dcu.ie (M.H.); paula.meleady@dcu.ie (P.M.); 3 School of Biotechnology, Dublin City University, Dublin 9, Dublin, Ireland

**Keywords:** milk, teramana goat, saanen goat, proteomics, label-free LC–MS

## Abstract

**Simple Summary:**

Milk is a valuable source of proteins and other nutrients. Changes in milk production toward systems that obtain increased milk yield have resulted in a change in milk composition in large and small ruminants, such as goats. A better characterisation of breeds that undergo a limited formal crossbreeding, such as the Teramana goat is pivotal in order to obtain useful data that could be applied for the management of the breed. Proteomic technologies have brought significant advances in the characteristion of new proteins. In this study, this technology was used to compare the proteome of the autochthon Teramana and Saanen breeds, which are commonly used by the industry to allow for the identification of a cohort of proteins that were able to discriminate the two goat breeds. Proteomics offered the potentiality for a deeper investigation of the biological differences of the breeds under study in a substrate easy to obtain.

**Abstract:**

Goat’s milk is an excellent source of nutrients, with greater benefits compared to cow’s milk. Limited information is available on autochthon goat breeds, which are important for biodiversity preservation. In this study, the aim of using label-free quantification was to investigate the milk proteome of two goat breeds, the autochthon Teramana and Saanen breeds, which are commonly used by the industry. Utilising label-free proteomic analysis, 749 and 666 proteins, respectively were identified and quantified from the Teramana and Saanen goat milk. Moreover, utilising statistical analysis, 29 proteins were able to discriminate the two goat breeds, with many of the identified proteins involved in complement and coagulation cascades. This work enhances our understanding of the goat milk proteome and shows differences between the two breeds, leading to an important contribution toward a more detailed molecular-view of this unique substrate. Additionally, charactersation of the milk proteins can help in guiding genetic improvements in the goat herds, and thus increasing its use in human nutrition.

## 1. Introduction

Milk plays a pivotal role in human nutrition, as it is rich in macro- and micro-nutrients and is an important source of antimicrobial and immunoregulatory agents. Over the last decades, the use of non-bovine milk as an alternative nutrient source has increased, since hypersensitivity to cow’s milk proteins remains one of the major causes of food allergies [1]. For this reason, the milk of small ruminants, such as goats is of particular interest to some consumers as it is easier to digest than milk from cows and may have certain therapeutic values [2]. The major differences between cow’s and goat’s milk are related to the different proportions and classes of caseins [3]. Other differences are the size and structure of the protein micelles and fat globules with a more favourable fat composition in goat’s milk, which is beneficial for digestibility and energy uptake, and makes the goat’s milk less allergenic [4,5,6,7]. Additionally, these properties are beneficial for the cheese manufacturer, as goat milk allows for the development of fermented products with particular characteristics compared to cow’s milk [8]. Moreover, goats are of particular economic interest in developing countries as they can easily adapt to harsh environmental conditions, have the capacity to convert poor quality fibrous feedstuffs into animal proteins, and have a high resistance to diseases, which make the production of this milk a useful strategy to tackle the problems of under-nutrition [9].

Despite the fact that the world goat population has increased during the last decades, many autochthonous breeds have been displaced and are in an endangered status. They even face extinction since they have been replaced by more productive breeds. This is the case for breeds, such as Teramana and Saanen goats. The Teramana goat is an autochthonous breed from the Abruzzo region of Italy, which as a consequence of the marked decrease in fresh goats milk consumption and of the progressive abandoning of low income rural activities by farmers, has become almost extinct [10]. Currently, and according to official national data reported by the Food and Agriculture Organisation of the United Nations (FAO), this breed is listed with a critical risk status. The Saanen goat is a highly productive dairy goat and is one of the most distributed goat breeds in many countries [11]. 

Proteomics consist of the high-throughput study of the proteome (the protein complement of a genome). The proteome is the entire set of proteins that is produced or modified by a cell, an organism, a body fluid, etc. at a given time under defined conditions [12,13]. Due to these features, proteomics is ideally suited to characterise and map the goat’s milk proteome. Proteomic technologies have brought significant advances in the detection and identification of new proteins in goat’s milk. For many years, gel-based proteomic approaches, such as SDS PAGE [14] and two-dimensional gel electrophoresis (2D PAGE) [15,16] were the main techniques used to investigate the protein composition of goat’s milk. Following, the development of mass spectrometry (MS)-based proteomic methods, such as label-free MS-based quantification has become more popular. This is a method for measuring peptide concentrations in complex samples using a combination of high-performance liquid chromatography (HPLC) and MS, which does not require expensive labelling techniques [17,18]. A variety of milk proteins have been identified by applying this approach with bioinformatic tools [2,19]. Moreover, in our previous study, this method has been successfully applied to discriminate the meat proteome of the Teramana and Saanen goat breeds [10]. 

Caseins make up 80% of overall protein content of milk [20] and their presence is a limiting factor in studying less abundant proteins that have also been proposed as possible biomarkers for animal health [21]. Prefractionation and further depletion of medium- to high-abundance proteins is required to study minor components [22,23]. A method, such as label-free LC–MS proteomics is ideal for these analyses in order to allow for the study of hydrophobic proteins and proteins with low- or high-molecular weights [17,18]. 

Studies on goat milk proteomics have mainly focused on the more productive goat breeds [24,25,26]. Interspecies differences can be evaluated using proteomic analysis; however, studies evaluating the milk proteome from different breeds of the same species, particularly among goat breeds, are still scarce [27]. In this context, studies with an emphasis on the comparative proteomic evaluation of goat milk can help in guiding genetic improvements in the goat herds, and thus increasing its use in human nutrition. Therefore, in this study, a label-free proteomic approach was applied to characterise the milk proteome of the Teramana and Saanen goat breeds, which may lead to a better understanding of the biological mechanism of this product.

## 2. Materials and Methods

### 2.1. Sample Collection

Five Teramana and five Saanen goats for a total of 10 animals aged 30 to 40 months with no signs of acute mastitis or other clinical diseases were selected from a local farm in Teramo province, Italy. All goats included in this study were reared in the same farm, and the management with regard to their feeding, handling, and period of lactation was the same. Thereafter, approximately 100 mL of milk was collected by milking at one time point from each animal and were used in the study. All animals were representative of goat breeds. After sampling, all milk samples were transferred on ice, and then taken immediately to the laboratory, where they were processed. No permits were required for the described study, which complied with all relevant regulations, since only milk was collected and no animal sacrifice was necessary.

### 2.2. Sample Preparation

Goat’s milk samples (2 mL) were centrifuged at 3000× *g* for 30 min, at 4 °C, and the fat layer was carefully removed. Then, 50 µL of the skim milk samples were precipitated by adding 5 µL of sodium acetate (1 N) and 5 µL of acetic acid (10%), and the caseins were removed by centrifugation at 3000× *g* for 30 min, at 4 °C. Whey proteins were directly precipitated with cold acetone, dried, and stored at −20 °C until further analysis.

The protein content of all samples was determined in triplicate using the Protein Assay Kit (Bio-Rad Labs, Hercules, CA, USA), following the Bradford method using a bovine serum albumin (BSA) standard [28]. Equal concentrations (100 μg) of all protein samples were used for filter-aided sample preparation (FASP) as described below [29].

### 2.3. Filter-Aided Sample Preparation (FASP)

Equal concentrations (100 μg) of all protein samples were purified and digested according to a slightly modified filter-aided sample preparation (FASP) method [29,30]. Briefly, 100 μg of milk proteins were suspended in 100 μL of lysis buffer [7M Urea (Affymetrix/Thermo Fisher Scientific, Waltham, MA, USA), 2M Thiourea (Affymetrix/Thermo Fisher Scientific, Waltham, MA, USA), 30 mM Tris, 4% CHAPS (Affymetrix/Thermo Fisher Scientific, Waltham, MA, USA), pH 8.5]. Protein samples were reduced and alkylated with dithiothreitol (DTT) and iodoacetamide (IAA; Sigma-Aldrich/Merck, Saint Louis, MO, USA), and then digested with trypsin according to the FASP method [29,30]. Peptides were purified using C18 spin columns (Thermo Fisher Scientific, Waltham, MA, USA), dried under vacuum, and suspended in 2% acetonitrile (ACN) and 0.1% trifluoroacetic acid (TFA) prior to mass spectrometry.

### 2.4. Mass Spectrometry for Label-Free LC–MS

Nano LC–MS/MS analysis was carried out using an Ultimate 3000 nanoRSLC system (Thermo Scientific) coupled in-line with an Orbitrap Fusion Tribrid™ mass spectrometer (Thermo Scientific). Then, 1 µg of digested protein samples were loaded onto a C18 trap column (C18 PepMap100, 300 μm × 5 mm, 5 μm particle size, 100 Ǻ pore size; Thermo Scientific) and desalted for 3 min using a flow rate of 25 μL/min in 0.1% (*v*/*v*) TFA, 2% (*v*/*v*) ACN as previously described by Di Luca et al. [10].

### 2.5. Label-Free LC–MS Quantitative Profiling

Proteome Discoverer v.2.2 (Thermo Fisher Scientific) with the Sequest HT algorithm and Percolator was used to achieve the proteins identification. MS files were searched against the UniProtKB-SwissProt *Capra hircus* database (downloaded in February 2020 and containing 32,490 sequences). The parameters set for protein identification were essentially as described in detail in our previous study in the same species [10].

Progenesis QI for Proteomics (version 2.0; Nonlinear Dynamics, a Waters company, Newcastle upon Tyne, UK) was used for quantitative label-free data analysis as already described [10]. The steps for the alignment, normalisation which use ratiometric data in log space, along with a median and mean absolute deviation outlier filtering approach, calculation of peptide abundance were as recommended by nonlinear dynamics (Waters^TM^; www.nonlinear.com) and as described in our previous study [10]. Only peptide ions with charge states +1, +2, and +3 were considered and re-imported back into Progenesis QI software for further analysis. Peptide identifications were imported into the Progenesis QI software and assigned to the matching features. Proteins were considered differentially expressed if they passed the following criteria: (1) ANOVA values with a cut off of *p* < 0.05, (2) proteins with ≥2 peptides matched, and (3) ≥1.5-fold difference in abundance.

### 2.6. Functional and Protein Network Analyses

Proteins identified in both groups were submitted to classification analysis using the PANTHER (Protein Analysis Through Evolutionary Relationships) database system, release 14.1 (http://www.pantherdb.org/) [31]. Default parameters were used to carry out the analysis for the categorisation into biological process.

Cytoscape (http://www.cytoscape.org/; accessed on 8 May 2020) [32] using the plug-in ClueGO (http://www.ici.upmc.fr/cluego/; accessed on 8 May 2020) [33] was used for the functional interpretation of the differentially expressed proteins. Gene Ontology (GO) Biological Process (BP) branch (May 2020) was used for the gene enrichment analysis. The parameters used were as described in a previous study [34]. Due to the insufficient protein annotation for goat species, the analysis made use of *Bos taurus* specific functional annotations. Default parameters were used for the other parameters. GO:BP terms with a Benjamini–Hochberg corrected *p*-value < 0.05 were considered statistically over-represented. The ClueGO plug-in which integrates and the KEGG pathway database (May 2020) were also used to separately create functionally organised pathway term networks.

In silico protein–protein interaction (PPI) analysis of the proteins that were differentially expressed between goat milk breeds was carried out using the Search Tool for the Retrieval of Interacting Genes/Proteins (STRING v.11) database (https://string-db.org/; accessed on 22 November 2022) [35] as already described [34]. *Bos taurus* specific interactome was used for the analysis due to insufficient protein annotation for goat species. In the analysis, interactions with high confidence (>0.7) STRING combined score were considered.

## 3. Results

### 3.1. Identification of Milk Proteins in Goat Breeds

**Figure 1:**
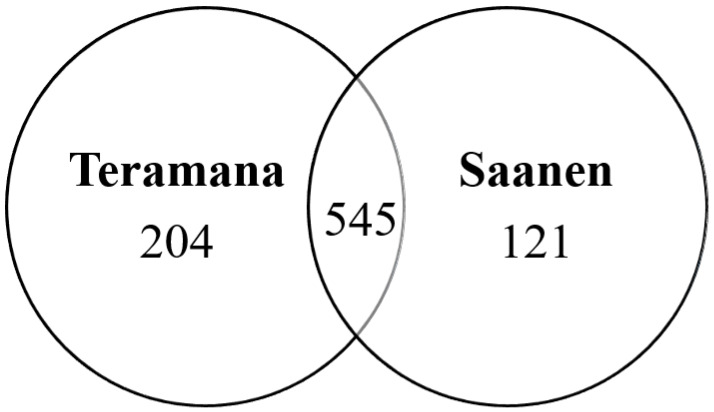
Venn diagrams showing the total number of proteins identified in the Teramana goat milk compared to Saanen goat milk. The full list of proteins are in Supplementary Material Tables S1 and S2.

In the present study, 749 (including 3349 unique peptides) and 666 (including 3057 unique peptides) proteins were identified from the Teramana and Saanen goat’s milk, respectively by label-free proteomic analysis (Supplementary Material Tables S1 and S2). Combining these two datasets, this study identified a total of 870 proteins. Among the proteins identified in the Teramana and Saanen goat milk breeds, respectively 204 (23.4%) and 121 (13.9%) proteins were unique to each breed and 545 (62.6%) proteins were common between the two breeds (Figure 1).

**Figure 2:**
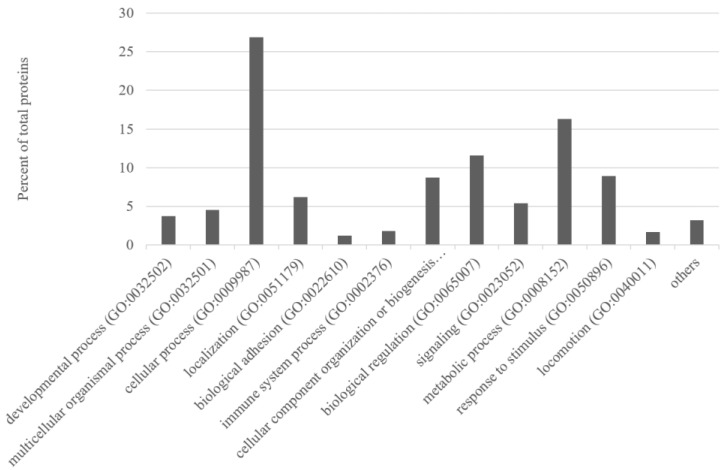
Percentage of milk proteins (over 870 identified proteins; Supplementary Material Tables S1 and S2) grouped according to different biological processes.

The ontology tools in PANTHER indicated that the majority of these proteins (870 proteins) were mainly involved in cellular process 26.9%, in metabolic process 16.3%, biological regulation 11.6%, response to stimulus 8.9%, and cellular component organisation or biogenesis 8.7% (Figure 2). The unique proteins identified in the Teramana were mainly involved in cellular process (29.4%), metabolic process (20%), biological regulation (11.3), cellular component organisation or biogenesis (9.1%), and response to stimulus (8.3%). Whereas the unique proteins identified in the Saanen were mainly involved in cellular process (26.4%), metabolic process (11.7%), biological regulation (11.7), response to stimulus (10.4%), cellular component organisation or biogenesis (8.6%), and signalling (8%) (Supplementary Material Figure S1).

### 3.2. Label-Free Quantitative Proteomic Analysis of Goat Milk

Label-free quantitative proteomics was used to compare goat milk samples from the Teramana and Saanen breeds. Differences at the proteome level between the two breeds were investigated using the label-free software, Progenesis QI for Proteomics. Normalised proteomics data were used to identify the differentially expressed proteins between the Teramana and Saanen breeds. Differentially expressed proteins were defined as those that showed a fold change cut-off greater than 1.5, a *p*-value ≤ 0.05 (one-way ANOVA), and a number of unique peptides greater than 2 that matched the protein. Based on these criteria, there were 29 differentially expressed proteins between the Teramana and Saanen goat milk breeds (Table 1), of which 18 (62.1%) were upregulated proteins in the Teramana breed and 11 (37.9%) were upregulated in the Saanen breed. The full list of the 29 proteins identified in this study is shown in Table 1.

### 3.3. Functional Association Analysis

**Figure 3:**
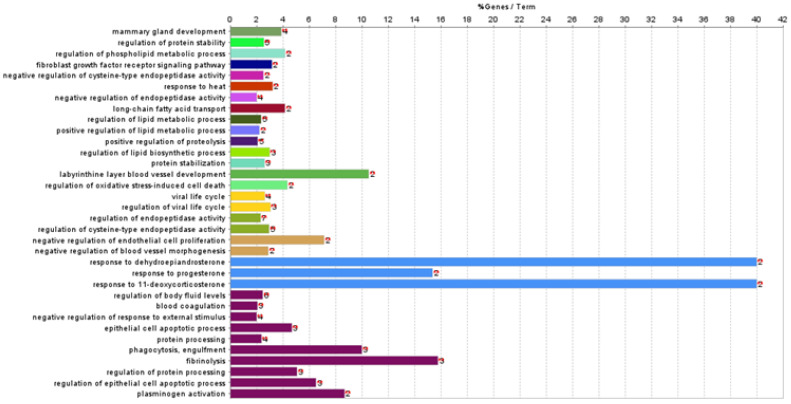
Gene enrichment analyses over the Gene Ontology—Biological Process branch of the 29 differentially abundant milk proteins between the Teramana and Saanen goat breeds. Bars represent the percentage of input proteins found associated with respect to the number of proteins directly annotated with the functional term. The term significance and the number of input proteins related to the term are reported next to each bar. Detailed statistics are reported in Table 2. In each panel, bars sharing a specific colour are clustered in the same functional group (Table 2).

Functional association analysis was used to investigate the biological processes and pathways involving the 29 differentially abundant proteins identified. Enrichment analyses were carried out in Cytoscape using the ClueGO plug-in. Analyses were run separately over the GO:BP and KEGG pathway databases. Three proteins (immunoglobulin heavy constant Gamma 4, IGHG4; Ig-like domain-containing protein, rig-5; and cysteine-rich secretory protein 3, Crisp3) were not in the ClueGO annotation sets. A total of 34 GO:BP terms (involving 18 differentially abundant proteins) were retrieved (Table 2, Figure 3). These biological processes can be generally summarised in the following groups: (i) Biological regulation, (ii) cellular and developmental process, (iii) multicellular organismal process, (iv) response to stimulus, (v) signalling.

**Figure 4:**
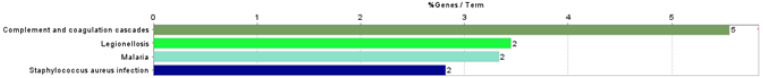
Gene enrichment analyses over the KEGG pathway database of the 29 differentially abundant milk proteins between the Teramana and Saanen goat breeds. Bars represent the percentage of input proteins found associated with respect to the number of proteins directly annotated with the functional term. The term significance and the number of input proteins related to the term are reported next to each bar. Detailed statistics are reported in Table 3.

Over-representation analysis using the KEGG pathway database highlighted a total of four pathways (Table 3, Figure 4) related to the response to stimulus. Eight proteins were involved in these pathways. The differentially abundant proteins involved in the complement and coagulation cascades, as previously shown from the analysis over the GO:BP database, are of higher abundance in the Teramana breed.

### 3.4. Protein–Protein Interaction (PPI) Analysis

**Figure 5:**
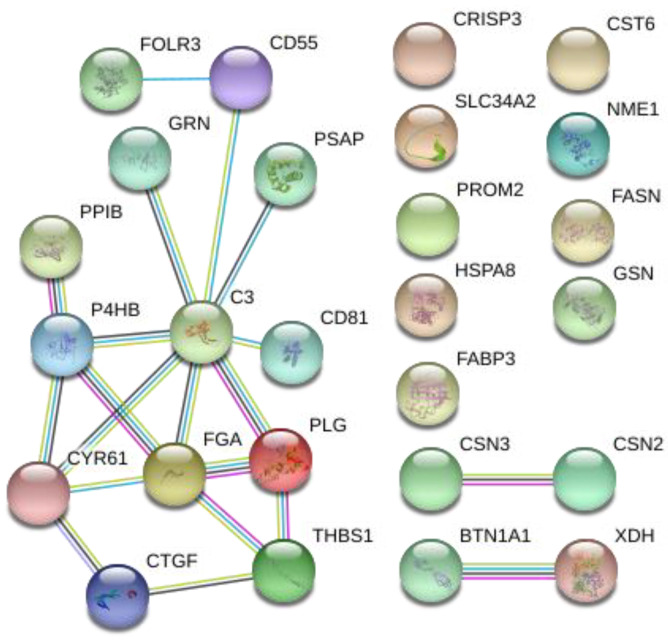
Protein–protein interaction (PPI) network of the 29 differentially abundant proteins in goat milk. Each node represents a protein, and different line colours represent the types of evidence for the association: Cyan is from curated databases, magenta is experimentally determined, dark green is gene neighborhood, blue is gene co-occurrence, light green is text-mining, black is co-expression, and purple is protein homology.

PPI analysis was performed using STRING with all the significantly different proteins identified in the comparison of two goat breeds (Figure 5). A connected protein network was revealed by the analysis. The protein network was composed of 26 nodes divided in: (i) One big module composed of thirteen nodes (50%), (ii) two small components of two proteins (15.4%), and (iii) nine singletons (34.6%). The resulting network showed a PPI enrichment *p*-value of 4.32 × 10^−8^ (four expected edges vs. twenty detected edges) indicating that proteins are at least partially biologically connected. In this network, most of the proteins interacted with only one or two other partners (average node degree equal to 1.54). Two proteins complement C3 (C3) and fibrinogen alpha chain (FGA) presented the highest degree of connection (eight and five edges, respectively), which may assign to them a role as “hub” proteins playing a putative function of controllers inside biochemical pathways that could potentially lead to cascade of protein expression differences. Both proteins had a higher expression in the Teramana goat breed. Moreover, most of the proteins clustered in the big module were included in the GO and KEGG enrichment processes clearly differentiating the two goat breeds.

## 4. Discussion

The advancement of electrophoresis and chromatography, along with developments in mass spectrometry technologies, have widened the potential application of proteomics to study milk proteomes from smaller ruminants, such as goats. Milk is an easily accessible body fluid rich in proteins, which are important for tissue growth and cellular functions. Moreover, some proteins can act as hormones, whereas others display antimicrobial properties [1]. These characteristics make milk a promising substrate to investigate proteins and peptides indicative of molecular processes underpinning differences between breeds. Furthermore, this substrate could be easily used for the identification of biomarkers that could enable a genetic improvement in the goat herds and/or to monitor diseases. The aim of the present study was to investigate and compare the whey proteome of the autochthonous Teramana and Saanen goat breeds.

In the last two decades, great efforts have been addressed to increase the study of milk proteomics, especially from bovine. In more recent times, the study of milk proteome from smaller ruminants, such as goat have received great interest, with whole milk, whey, milk fat globule membrane (MFGM) fractions, and casein fractions as the main substrate utilised [2,14,15,16,19,36]. A study of Chen et al. [37] identified a total of 843 proteins by comparing the proteome of goat milk during heated processing using label-free quantification. Using a similar approach, 595 and 486 proteins were characterised in the whey of two autochthon Greek goat breeds [27].

In our study, the label-free LC–MS analysis revealed a total of 870 different proteins in the two goat breeds (Supplementary Material Tables S1 and S2) under study. The comparable and higher number of proteins identified in this study indicates the successful use of proteomic techniques. Many of the identified proteins (e.g., complement C3, fibronectin, plasminogen, calcium and integrin binding 1) were also identified in other studies [26,27,36]. Most of the identified proteins were involved in cellular and metabolic process, biological regulation, response to stimuli, etc. that characterise milk (Figure 2). Comparing the unique proteins identified in each breed, the Teramana breed show a higher number of proteins involved in metabolic process (Supplementary Material Figure S1). A similar class of proteins was also identified in whey from Greek goat and sheep breeds [27]. Furthermore, results are in agreement with those presented in the study of Cunsolo et al. [38], who investigated the goat milk proteome in the Camosciata goat breed.

This work is the first study that applies a label-free method to unravel the milk whey proteome of the Teramana and Saanen goat breeds. Whey proteins show specific characteristics, which reflect the physiological requirements of the animal [1]. Utilising statistical analysis, 29 proteins were differentially expressed between the two breeds, highlighting interspecies difference. To our knowledge, this is the first study to observe proteins that are changing significantly in whey between goat breeds. Using the same method, Zhao al. [39] identified 156 differential MFGM proteins between yak and cow. The higher number of proteins identified in their study compared to ours is mainly due to the different species used for the comparison. As expected, the dominant classes of proteins identified in our substrate were whey proteins; however, residues of caseins were also observed.

Among the 29 proteins changing significantly in milk between the two goat breeds, the top differentially expressed proteins (complement C3, C3; fibrinogen alpha chain, FGA; cellular communication network factor 1, CYR61; plasminogen, PLG; and protein disulfide-isomerase, P4HB) according to their functions, may contribute to explaining in part the phenotypic differences between the Teramana and Saanen goat breeds. Functional analysis of the proteins identified in our study highlights their role in a wide range of regulatory processes, such as lipid metabolic process, regulation of proteolysis, oxidative stress-induced cell death, etc. KEGG pathway shows that most of the whey proteins upregulated in the Teramana breed are involved in complement and coagulation cascades, whereas three pathways were involved in disease-related pathways in both breeds. The complement and coagulation cascades pathway plays an important role in activating innate immunity, in maintaining the balance of the coagulation-fibrinolytic system, and the associated proteins are critical for the health and nutrition of goat kids [40,41,42]. The upregulation observed in the Teramana breed may allude to a greater capacity of this autochthon breed to adapt to harsh environments compared to breeds that are increasingly used from the industry. Whey proteins involved in the same pathways were also highlighted in a study by Sun at al. [26] on the colostrum and mature milk of Xinong Saanen goats.

Complement C3 (C3) was upregulated in the Teramana goat. Complement is a central component of the innate immune system. Its main functions include host defence against agents, facilitating adaptive immune responses and elimination of immune complexes and apoptotic cells [43]. The complement system consists of about 35/40 proteins, which are usually associated with blood cells and blood plasma, but found generally at lower concentrations in other secretions of the body-like milk [44]. Difference of C3 level between Teramana and Saanen breeds might be in-line with a higher defence mechanism of the goat mammary gland against infections of the first breed. It has been shown that the concentration of C3 is significantly higher in preterm human milk than term human milk and it has been postulated that this may be due to the higher requirement of protection from the infants in its early days, as the immune system is less developed than term infants [45]. Similar mechanisms may occur in goat breeds, such as the Teramana that are usually keener to live in harsh environments. These animals are more exposed to pathogens, and thus require a higher protection. Fibrinogen (FGA) is a complex plasma protein required for the last phase of blood coagulation [46]. FGA is one of the acute phase proteins (APPs) which are a serum component whose concentrations vary under external or internal influences, such as inflammation, stress, etc. These proteins are important early diagnostic markers of inflammation in animals [47]. In our study, an upregulation of fibrinogen alpha chain was observed in the Teramana breed whey. It has been shown that the main agent involved in mastitis is *Staphylococcus aureus* and that molecules, such as fibrinogen can change in abundance during infection [48]. The animals used in this study did not show any signs of mastitis or other clinical diseases and the high concentration of APPs is not always a sign of disease [49], the upregulation of this protein may allude to a stronger resistance of the Teramana breed (namely, in general more rustic compared to commercial breeds) to this kind of disease. Cellular communication network factor 1 (CYR61) and cellular communication network factor 2 (CTGF) (upregulated in the Teramana breed) are cysteine-rich proteins of the CCN family. These proteins mediate many functions, such as cell survival and apoptosis [50,51]. In mammals, a reduced suckling frequency initiates the downregulation of milk synthesis and the induction of apoptotic pathways and structural remodelling of mammary tissue with a corresponding reduction in secretory activity [52]. It has been proposed that CYR61 and CTGF proteins promote apoptosis of mammary epithelial cells, and that their presence points to the mechanisms underlying the lactation inhibition [53]. It is known that the milk production of the Teramana breed is lower compared to the Saanen [11]. The upregulation of these proteins observed in our study in the Teramana breed may suggest a role played by these proteins in the mechanism involved in the lower milk production of the Teramana breed and highlight the potentiality of these proteins as markers to discriminate between the milk of the two breeds. Plasmin is one of the major endogenous protease present in milk that is secreted in its inactive form plasminogen (PLG), which is the predominant form in fresh milk [54]. Plasmin is then activated and inhibited by the levels of plasminogen activators and inhibitors [55]. Theodorou et al. [56] highlighted that among the factors affecting the plasmin-plasminogen system on sheep’s milk, there is the breed factor. Milk contains activins of plasminogen, which can activate fibrinolysis and keep the secretion pipeline unobstructed [26]. In our study, PLG was upregulated in the Teramana breed, which may allude to a stronger resistance of milk duct infections of this breed, namely, in general considered more rustic compared to commercial goat breeds. Moreover, plasmin plays a pivotal role in cheese ripening by breaking down α- and β-caseins to result in flavour and texture development [57]. On the other hand, the activity of this protein has been associated with alteration of mammary epithelium permeability and with an increment in paracellular flow, activities that aggravate the milk quality, coagulation properties, and cheese yield. The presence of mastitis, the increment of the age of the animals, and stage of lactation increase the level of PLG [58]. As the animals used in our study had no signs of mastitis or other clinical diseases and were of a similar age, the higher abundance of PLG observed in the Teramana breed may contribute to the development of a characteristic flavour and texture of the dairy products made by the milk of this autochthon breed. Protein disulfide isomerase (P4HB) is a member of the thioredoxin superfamily of redox proteins that is mainly located in the endoplasmic reticulum. P4HB has multiple roles, is a binding partner of other proteins, acts as a chaperone, is a hormone reservoir, as well as a disulfide isomerase in the formation of disulfide bonds [59]. Given their pivotal role in protein-folding, the loss of P4HB activity and the consequent accumulation of misfolded proteins have been associated with the pathogenesis of numerous disease states [60]. To our knowledge, no other studies observed changes in this protein among dairy breeds, the upregulation observed in the Teramana breed might allude to a stronger resistance to disease of this breed that undergo a limited formal crossbreeding. Indeed, due to the negative genetic correlation evidenced between milk production traits and health traits, the genetic selection for higher milk production has been associated with an increased sensitivity to diseases [61,62].

## 5. Conclusions

In summary, using label-free proteomics, it was possible to highlight proteomic differences between the whey fraction of the milk from the autochthon Teramana goat breed and Saanen goat breed. Casein and whey proteins are the major proteins of milk, where the casein constitutes approximately 80% of the total protein. An in-depth characterisation of both fractions will benefit dairy productions, will contribute to the conservation on animal biodiversity, and will help in the design of novel products. In this study, we report that the most extensive investigation on the goat whey proteome of a total of 870 proteins was identified. Twenty-nine proteins were able to discriminate between Teramana and Saanen goat breeds with many of the identified proteins involved in complement and coagulation cascades. These findings elucidate the proteome composition of milk whey and quantitative protein profile in the analysed goat’s milk. Moreover, the association between the proteins and milk from the two breeds supports the potential use of proteomic profile as a predictive biomarker in milk substrate that can discriminate between different genetic backgrounds. This may be used to evaluate milk adulteration of specific milk and expand the potential direction for the production of specific milk proteins.

## Figures and Tables

**Table 1:** Twenty-nine proteins identified as differentially expressed in goat milk between the Teramana and Saanen breeds following label-free MS/MS analysis (Progenesis QI for proteomics). Proteins are ordered according to the number of peptides identified.

UniProt *	Gene Name	Description	Peptides ^§^	Score ^¥^	ANOVA (*p*)	Fold Change	Highest Condition ^ǂ^
A0A452DX18	C3	Complement C3	27	94.92	0.0097	4.03	Teramana
A0A452F1G2	THBS1	Thrombospondin 1	9	24.44	0.0007	24.62	Teramana
A0A452DRX5	PSAP	Prosaposin	6	18.88	0.0031	2.63	Teramana
A0A452FHJ7	GSN	Gelsolin	6	20.57	0.0056	1.52	Teramana
A0A452E7A0	PLG	Plasminogen	6	22.23	0.0241	2.27	Teramana
A0A452FK23	CSN2	Beta-casein	4	10.73	0.0086	2.06	Teramana
A0A452G9D9	CSN3	Kappa-casein	4	36.55	0.0140	2.53	Teramana
A0A452E2Y0	CLU	Clusterin	4	15.79	0.0292	4.47	Teramana
D6PX62	Crisp3	Cysteine-rich secretory protein 3	3	9.86	0.0026	38.63	Teramana
A0A452G7V5	P4HB	Protein disulfide-isomerase	3	9.50	0.0245	2.12	Teramana
A0A452EZB5	CD55	Complement decay-accelerating factor	3	7.99	0.0267	3.89	Teramana
A0A452F9Y8	PPIB	Peptidyl-prolyl cis-trans isomerase	3	10.26	0.0341	1.98	Teramana
A0A452GBG4	CYR61	Cellular communication network factor 1	2	7.98	0.0001	23.17	Teramana
A0A452EKN2	IGHG4	Immunoglobulin heavy constant gamma 4	2	6.11	0.0006	9.56	Teramana
A0A452DZD6	rig-5	Ig-like domain-containing protein	2	8.56	0.0029	1.51	Teramana
A0A452G5W1	GRN	Granulin precursor	2	6.71	0.0034	3.02	Teramana
A0A452F4L3	FGA	Fibrinogen alpha chain	2	5.47	0.0194	2.46	Teramana
A0A452G077	CTGF	Cellular communication network factor 2	2	6.67	0.0402	6.69	Teramana
A0A452EZW6	XDH	FAD-binding PCMH-type domain-containing protein	14	38.58	0.0089	1.96	Saanen
A0A452EYF6	HSPA8	Heat shock cognate 71 kDa protein	5	15.53	0.0011	1.72	Saanen
A0A452ERT5	SLC34A2	Solute carrier family 34 member 2	4	9.43	0.0002	5.17	Saanen
A0A452E4K0	CST6	Cystatin E/M	4	15.21	0.0041	2.86	Saanen
Q6S4N9	FABP3	Fatty acid binding protein 3	3	7.85	0.0002	1.75	Saanen
A0A452FN55	BTN1A1	Butyrophilin subfamily 1 member A1	3	8.43	0.0058	1.76	Saanen
A0A452F4U3	FASN	Fatty acid synthase	3	10.66	0.0104	4.12	Saanen
A0A452G9E6	FOLR3	Folate_rec domain-containing protein	3	8.19	0.0164	1.83	Saanen
A0A452F3X9	PROM2	Prominin 2	2	5.84	0.0116	2.99	Saanen
A0A452E0U0	CD81	Tetraspanin	2	13.07	0.0211	2.28	Saanen
A0A452F230	NME1	Nucleoside diphosphate kinase	2	4.55	0.0320	1.52	Saanen

Eighteen proteins were upregulated in milk from the Teramana breed and eleven proteins were upregulated in milk from Saanen goat breed. (*) Accession number in the UniProt database; (^§^) unique peptides used for quantitation; (^¥^) SEQUEST score. (^ǂ^) Indicates whether the proteins were upregulated in the Teramana or in the Saanen goat breeds.

**Table 2:** Over-represented biological processes (GO:BP) associated with upregulated or downregulated proteins in the goat milk breed comparison.

GOID	Description	Functional Group ^1^	*p*-Value ^2^	% of Associated Proteins ^3^	No. of Proteins	Upregulated or Downregulated Proteins ^4^
GO:0030879	mammary gland development	G0	0.000057	3.88	4	CSN2 ↓, CSN3 ↓, FASN ↑, XDH ↑
GO:0031647	regulation of protein stability	G1	0.000053	2.56	5	CD81 ↑, CLU ↓, CSN3 ↓, GSN ↓, PPIB ↓,
GO:1903725	regulation of phospholipid metabolic process	G2	0.003120	4.17	2	CD81 ↑, FABP3 ↑
GO:0008543	fibroblast growth factor receptor signalling pathway	G3	0.004663	3.17	2	CTGF ↓, THBS1 ↓
GO:2000117	negative regulation of cysteine-type endopeptidase activity	G4	0.006824	2.53	2	CSN2 ↓, THBS1 ↓
GO:0009408	response to heat	G5	0.004820	3.23	2	CSN2 ↓, PLG ↓
GO:0010951	negative regulation of endopeptidase activity	G6	0.000418	2.00	4	C3 ↓, CSN2 ↓, CST6 ↑, THBS1 ↓
GO:0015909	long-chain fatty acid transport	G7	0.003120	4.17	2	FABP3 ↑, THBS1 ↓
GO:0019216	regulation of lipid metabolic process	G8	0.000060	2.36	5	C3 ↓, CD81 ↑, CYR61 ↓, FABP3 ↑, PSAP ↓
GO:0045834	positive regulation of lipid metabolic process	G9	0.007858	2.22	2	CD81 ↑, CYR61 ↓
GO:0045862	positive regulation of proteolysis	G10	0.000071	2.09	5	C3 ↓, CLU ↓, CYR61 ↓, GSN ↓, XDH ↑
GO:0046890	regulation of lipid biosynthetic process	G11	0.000733	3.00	3	C3 ↓, CYR61 ↓, FABP3 ↑
GO:0050821	protein stabilisation	G12	0.001011	2.61	3	CLU ↓, CSN3 ↓, PPIB ↓
GO:0060716	labyrinthine layer blood vessel development	G13	0.000680	10.53	2	CYR61 ↓, PLG ↓
GO:1903201	regulation of oxidative stress-induced cell death	G14	0.003088	4.35	2	P4HB ↓, PSAP ↓
GO:0019058	viral life cycle	G15	0.000186	2.63	4	CD81 ↑, GSN ↓, P4HB ↓, PPIB ↓
GO:1903900	regulation of viral life cycle	G15	0.000186	3.06	3	GSN ↓, P4HB ↓, PPIB ↓
GO:0052548	regulation of endopeptidase activity	G16	0.000004	2.31	7	C3 ↓, CSN2 ↓, CST6 ↑, CYR61 ↓, GSN ↓, THBS1 ↓, XDH ↑
GO:2000116	regulation of cysteine-type endopeptidase activity	G16	0.000004	2.96	5	CSN2 ↓, CYR61 ↓, GSN ↓, THBS1 ↓, XDH ↑
GO:0001937	negative regulation of endothelial cell proliferation	G17	0.006928	7.14	2	THBS1 ↓, XDH ↑
GO:2000181	negative regulation of blood vessel morphogenesis	G17	0.006928	2.90	2	THBS1 ↓, XDH ↑
GO:1903494	response to dehydroepiandrosterone	G18	0.000390	40.00	2	CSN2 ↓, CSN3 ↓
GO:0032570	response to progesterone	G18	0.000390	15.38	2	CSN2 ↓, CSN3 ↓
GO:1903496	response to 11-deoxycorticosterone	G18	0.000390	40.00	2	CSN2 ↓, CSN3 ↓
GO:0050878	regulation of body fluid levels	G19	0.000014	2.48	6	CSN2 ↓, CSN3 ↓, FGA ↓, PLG ↓, THBS1 ↓, XDH ↑
GO:0007596	blood coagulation	G19	0.000014	2.07	3	FGA ↓, PLG ↓, THBS1 ↓
GO:0032102	negative regulation of response to external stimulus	G19	0.000014	2.00	4	CSN2 ↓, FGA ↓, PLG ↓, THBS1 ↓
GO:1904019	epithelial cell apoptotic process	G19	0.000014	4.69	3	FGA ↓, GSN ↓, THBS1 ↓
GO:0016485	protein processing	G19	0.000014	2.38	4	C3 ↓, FGA ↓, GSN ↓, THBS1 ↓
GO:0006911	phagocytosis, engulfment	G19	0.000014	10.00	3	C3 ↓, GSN ↓, THBS1 ↓
GO:0042730	fibrinolysis	G19	0.000014	15.79	3	FGA ↓, PLG ↓, THBS1 ↓
GO:0070613	regulation of protein processing	G19	0.000014	5.08	3	C3 ↓, GSN ↓, THBS1 ↓
GO:1904035	regulation of epithelial cell apoptotic process	G19	0.000014	6.52	3	FGA ↓, GSN ↓, THBS1 ↓
GO:0031639	plasminogen activation	G19	0.000014	8.70	2	FGA ↓, THBS1 ↓

^1^ Groups of closely-related terms; ^2^ Benjamini–Hochberg corrected *p*-values; ^3^ percentage of input proteins found associated with respect to the number of proteins directly annotated with the functional term; ^4^ the symbol ↑ indicates protein abundance higher in Saanen goat milk than Teramana goat milk, while the symbol ↓ indicates protein abundance higher in Teramana goat milk than Saanen goat milk.

**Table 3:** Over-represented KEGG pathways associated with upregulated or downregulated proteins in the goat breed milk comparison.

GOID	Description	Functional Group ^1^	*p*-Value ^2^	% of Associated Proteins ^3^	No. of Proteins	Upregulated or Downregulated Proteins ^4^
KEGG:04610	Complement and coagulation cascades	G0	0.000004	5.56	5	C3 ↓, CD55 ↓, CLU ↓, FGA ↓, PLG ↓
KEGG:05134	Legionellosis	G1	0.013192	3.45	2	C3 ↓, HSPA8 ↑
KEGG:05144	Malaria	G2	0.009393	3.33	2	CD81 ↑, THBS1 ↓
KEGG:05150	*Staphylococcus aureus* infection	G3	0.009755	2.82	2	C3 ↓, PLG ↓

^1^ Groups of closely-related terms; ^2^ Benjamini–Hochberg corrected *p*-values; ^3^ percentage of input proteins found associated with respect to the number of proteins directly annotated with the functional term; ^4^ the symbol ↑ indicates protein abundance higher in Saanen goat milk than Teramana goat milk, while the symbol ↓ indicates protein abundance higher in Teramana goat milk than Saanen goat milk.

## Data Availability

Not applicable.

## Supplementary Materials

The following supporting information can be downloaded at: https://www.mdpi.com/article/10.3390/ani13142263/s1. Table S1: Full list of the 749 proteins identified by mass spectrometry in the Teramana goat milk determined in Proteome Discoverer using SEQUEST HT algorithm. Table S2: Full list of the 666 proteins identified by mass spectrometry in the Saanen goat milk determined in Proteome Discoverer using SEQUEST HT algorithm. Figure S1: Percentage of milk proteins (over the unique proteins identified in the Teramana and Saanen goat breeds) grouped according to different biological processes.
