# Supplementary figures and images for: Comparative Label-Free Liquid Chromatography–Mass Spectrometry Milk Proteomic Profiles Highlight Putative Differences between the Autochthon Teramana and Saanen Goat Breeds
Source: Animals (Basel). 2023 Jul 10;13(14):2263. doi: 10.3390/ani13142263 (PMC10376190; doi:10.3390/ani13142263)

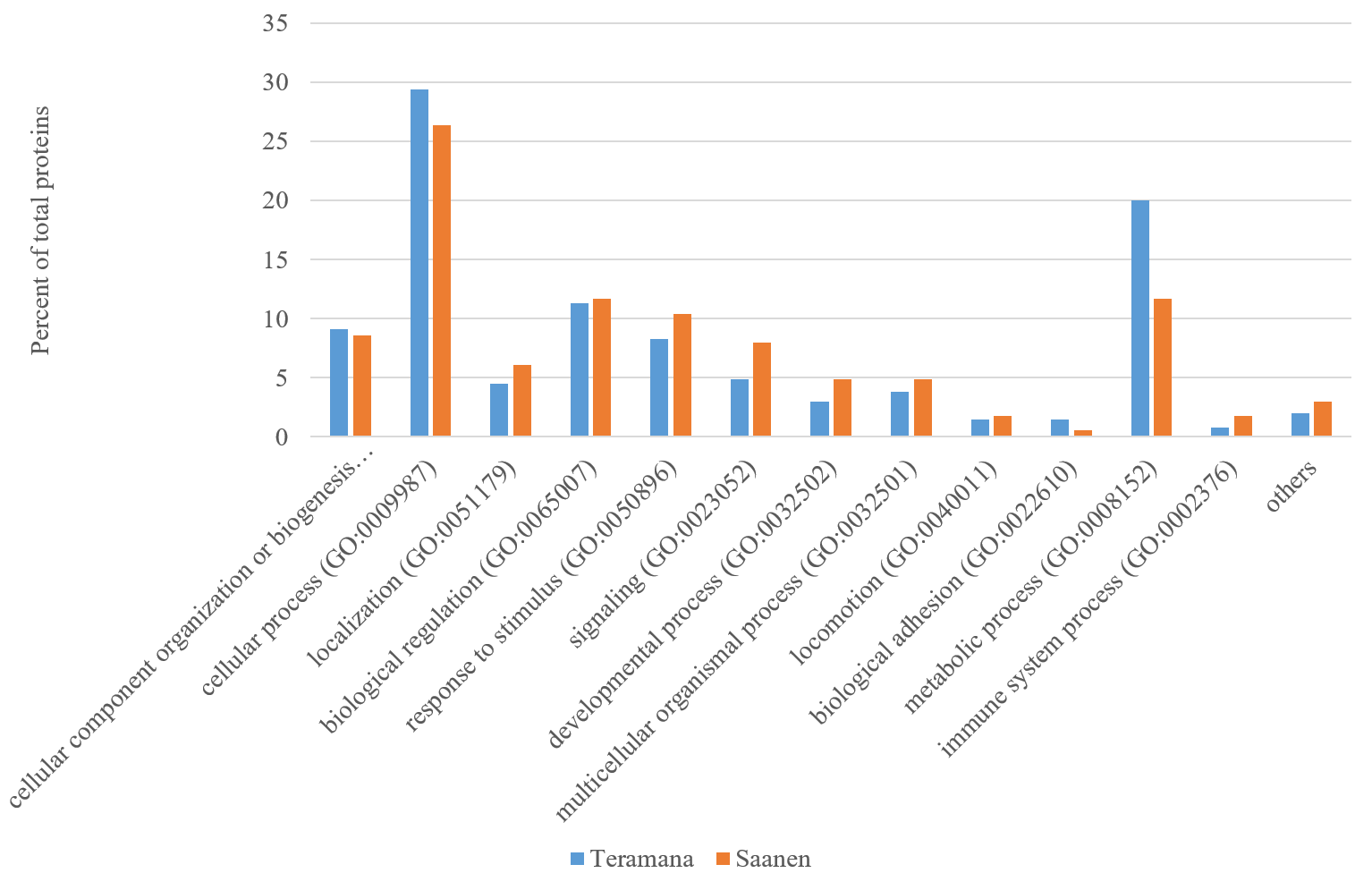

Supplement: Supplementary file 1 [file animals-13-02263-s001.zip › Figure S1.tif]
